# The Impact of Frailty, Activity of Daily Living, and Malnutrition on Mortality in Older Adults with Cognitive Impairment and Dementia

**DOI:** 10.3390/nu17162612

**Published:** 2025-08-12

**Authors:** Zitong Wang, Ying-Qiu Dong, Shikha Kumari, Diarmuid Murphy, Reshma Aziz Merchant

**Affiliations:** 1 Department of Biomedical Informatics, Yong Loo Lin School of Medicine, National University of Singapore, Singapore 119077, Singapore; wangzitong1638@163.com; 2 The Value Driven Outcome Office, National University Health System, Singapore 119228, Singaporeshikha_kumari@nuhs.edu.sg (S.K.); diarmuid_murphy@nuhs.edu.sg (D.M.); 3 Department of Orthopaedic Surgery, National University Hospital, Singapore 119074, Singapore; 4 Division of Geriatric Medicine, Department of Medicine, National University Hospital, Singapore 119228, Singapore; 5 Department of Medicine, Yong Loo Lin School of Medicine, National University of Singapore, Singapore 117566, Singapore

**Keywords:** dementia, frailty, activities of daily living, malnutrition, mortality, aging

## Abstract

Background: Malnutrition contributes to frailty dementia, intensifying adverse health outcomes including mortality risk. Objectives: We aim to investigate the impact of malnutrition risk in those with frailty and functional decline on short-term mortality among older adults with dementia and/or cognitive impairment. Methods: We conducted a retrospective cohort study involving 2677 hospitalized patients aged ≥65 years with a diagnosis of dementia or cognitive impairment discharged between March 2022 and December 2023. Information was obtained from electronic medical records. Frailty was assessed using the Clinical Frailty Scale (CFS) and Hospital Frailty Risk Score (HFRS), functional status using premorbid activity of daily living (ADL) scores, and malnutritional risk using the 3-Minute Nutrition Screening (3-Min NS) tool. Associations with 30- and 90-day mortality were examined using Kaplan–Meier analysis and multivariate logistic regression models. Results: A total of 29.2% were at risk of malnutrition, highest in the old-old (37.1%). Thirty-day mortality was significantly associated with CFS (aOR = 1.498, 95% CI: 1.349–1.664, *p* < 0.001), HFRS (aOR = 1.020, 95% CI: 1.001–1.040, *p* = 0.038), and ADL (aOR = 0.819, 95% CI: 0.753–0.890, *p* < 0.001). Malnutrition risk demonstrated the strongest association across all models (ADL: aOR = 2.573, 95% CI: 1.922–3.443, *p* < 0.001; CFS: aOR = 2.348, 95% CI: 1.738–3.156, *p* < 0.001; HFRS: aOR = 2.944, 95% CI: 2.210–3.922, *p* < 0.001). Associations between 90-day mortality and malnutrition risk remained significant across all models, including those adjusted for CFS and ADL. Notably, interactions between malnutrition and CFS further amplified mortality risk among the old-old (30-day: aOR = 1.435, 95% CI: 1.082–1.902, *p* = 0.012; 90-day: aOR = 1.263, 95% CI: 1.005–1.588, *p* = 0.045). Conclusions: Risk of malnutrition independently predicted short-term mortality in older adults with dementia or cognitive impairment, particularly among those with frailty, functional decline, and of advanced age.

## 1. Introduction

The global population is aging at an unprecedented pace. Advances in healthcare and improved living conditions have significantly increased life expectancy. This demographic transition is paralleled by an increasing burden of non-communicable diseases, such as hypertension, diabetes, dyslipidemia, dementia, and frailty. Both dementia and frailty are major medical and public health concerns that contribute significantly to the widening gap between health span and lifespan, often through prolonged periods of disability and dependency [1]. Dementia encompasses a range of neurological disorders with varied etiologies, and Alzheimer’s disease is recognized as one of the most common forms. It is marked by a progressive decline in cognition and activities of daily living, advancing to severe disability and mortality [1]. The prevalence of dementia in Singapore is 8.8% in ≥60 years old [2]. The number of persons living with dementia globally is estimated to be 152.8 million in 2050 [3]. In Asia, the prevalence has increased by 250.44% between 1990 and 2021 [4]. It is the seventh-most common cause of death globally [5].

Frailty is a dynamic state characterized by decreased physiological reserve, which predisposes the older individual to adverse outcomes when exposed to stressors [6]. In Singapore, studies show that about half of community-dwelling adults aged 80 years and older may have underlying frailty [7]. Frailty is categorized into physical, cognitive, and social domains, and it is a well-recognized risk factor for dementia [8]. It can accelerate the onset of dementia in at-risk individuals and has been shown to be reversible before the onset of disability [9,10].

Frailty and dementia share a bidirectional relationship, and their co-existence accelerates both cognitive and functional decline. This combination is further associated with poor quality of life, falls, institutionalization, and mortality [11,12,13]. The growing number of older adults affected by both frailty and dementia highlights the urgency for health policymakers to implement early intervention and improve healthcare strategies to support these vulnerable individuals and mitigate their declining health trajectories [14].

Activities of daily living (ADL) impairment is associated with poor quality of life, and important prognostic factor for mortality independent of underlying comorbid conditions [15,16]. In individuals with dementia, ADL decline captures both cognitive and physical deterioration and may serve as a practical surrogate for disease progression [7,17]. For instance, Stineman et al. reported a fivefold increase in one-year mortality among those who were completely dependent in their ADLs [15].

The burden of ADL impairment is substantial. In Singapore, one in ten community-dwelling older adults experiences at least one ADL impairment [7]. In the United States, the number of older adults with three or more ADL impairments is projected to rise between 4.5 and 7.4 million [15]. These trends highlight the growing prevalence of ADL impairment in the aging population. Although both dementia and frailty have a dwindling health decline, ADL impairment serves as a powerful, observable marker that captures the overlapping trajectories of physical and cognitive deterioration. Declining ADL may thus play a critical role in identifying individuals at risk of short-term mortality [18].

Malnutrition accelerates frailty and dementia through mechanisms such as muscle mass loss, immune dysregulation, altered gut microbiodata, and systemic inflammation [19]. In individuals with dementia and frailty, poor intake and gastro-intestinal changes can further disrupt the gut–brain axis, creating a vicious cycle of declining functional ability. Systematic reviews and meta-analyses indicate that the prevalence of frailty or pre-frailty among hospitalized older adults can reach 84%, while malnutrition or risk of malnutrition affects 66%, with nearly half of the conditions co-existing [20]. Malnutrition not only contributes to but also results from dementia, frailty, and ADL decline [21,22]. Impaired cognition and function can lead to inappropriate eating behaviors, loss of appetite [23], disordered eating patterns, and impaired access to food [24], which can further result in a downward spiral [25]. In addition, malnutrition increases vulnerability to adverse outcomes such as falls, prolonged length of stay, and increased healthcare cost [26]. Studies show that malnourished older adults aged ≥60 years have a threefold increased risk of developing dementia, twice the risk of ADL dependency, and a significantly higher short-term mortality risk [21,22,27].

Despite the well-established individual impact of frailty, ADL impairment, and malnutrition on adverse outcomes, most existing studies have focused on the general older population [28,29,30,31]. Although frailty and malnutrition have been identified as independent predictors of poor outcomes, the potential interaction effects, such as if malnutrition amplifies the mortality risk associated with frailty and ADL decline, represent an understudied area, particularly among older adults with dementia who have limited physiological reserve. Understanding the factors associated with short-term mortality is important in the era of rising healthcare costs for timely care planning and resource allocation. We aim to investigate the impact of malnutrition risk in those with frailty and functional decline on short-term mortality among older adults with dementia and/or cognitive impairment.

## 2. Method

### 2.1. Study Setting and Cohort of Analysis

A retrospective cohort study was conducted on older patients ≥ 65 years with either primary or secondary diagnosis of dementia and/or cognitive impairment obtained from electronic medical records. The diagnosis code and search strategies are described by Merchant et al. [32]. Both diagnoses (dementia and cognitive impairment) were included as DSM IV diagnostic criteria for dementia, requiring the individual to be out of delirium before a diagnosis can be made, despite a clinical history of possible dementia [33]. Data on 4238 in-hospital admissions at National University Hospital Singapore between March 2022 and December 2023 were collected but only data on 2677 admissions with complete records of Clinical Frailty Scale (CFS), Hospital Frailty Risk Score (HFRS), ADL, and 3-Minute Nutrition Screening (3-MinNS) were included in the final analysis.

### 2.2. Data Collection and Definition of Key Variables

Data on demographics, including chronic diseases, age, sex, HFRS, CFS, age-adjusted Charlson Comorbidity Index (CCI), ADL, and malnutrition risk, were obtained from the hospital administrative database. Assessment on premorbid CFS, i.e., prior to the onset of acute illness, was done at the triage area in the emergency department [34]. CFS 1–4 were categorized under robust/vulnerable, 5–6 mild/moderate, 7–8 as severe frailty, and 9 as terminally ill. HFRS was developed by Gilbert et al. in 2018 and calculated based on International Statistical Classification of Diseases and Related Health Problems, 10th Revision (ICD-10) codes [35]. It has since been validated globally and is associated with mortality and length of stay [36,37]. The HFRS score < 5 is considered low risk, 5–15 intermediate risk, and >15 high risk.

ADL was evaluated by nurses on admission and data were obtained for ADL two weeks prior to admission for six domains: bathing, dressing, toileting, transferring, continence, and feeding [38]. In this study, ADL independency score was defined as the number of activities patients were able to complete independently, ranging from “5–full independency” to “0–full dependency”. The 3-MinNS tool was used to screen for risk of malnutrition [39]. With a maximum score of 9, a score of ≥3 was used to define being at risk of malnutrition.

### 2.3. Outcomes

Thirty- and ninety-day mortality were measured as outcome variables for the study, representing short-term and medium-term impact of frailty, ADL, and malnutrition in older patients with dementia and/or cognitive impairment.

### 2.4. Statistics Methods

Descriptive statistics were used to summarize the demographic and clinical characteristics of participants, stratified by age (65–84, 85+ years old) and malnutrition status. Continuous variables were presented as means and standard deviations (SD), while categorical variables were expressed as counts and percentages. Comparisons between the two groups were performed using Chi-square ($\chi^{2}$) tests for both continuous variables (anaed as categories) and categorical variables. A two-sided *p*-value of 0.05 was used to indicate statistical significance. Kaplan–Meier survival curves were adopted to compare the differences in survival times based on groups of CFS, HFRS, ADL, and malnutrition. The log-rank test was performed to compare survival distributions among subgroups for each frailty index.

A multi-stage strategy was adopted for building the multivariate logistic regression models. First, three separate models were constructed by including only CFS, HFRS, or ADL, respectively. Next, malnutrition was added to each of these models to examine its incremental effect. The regression models were adjusted for CCI and baseline demographic characteristics. Finally, interaction terms for CFS, HFRS, ADL, and malnutrition were introduced into each of the three models to test for multiplicative effects.

To avoid potential bias resulted from multiple hospitalizations of the same patient, sensitivity analysis was conducted to test whether analysis using only the first-time hospitalization sample would affect the robustness of the results (available in the Supplementary Tables). In addition, sensitivity analyses were conducted to compare complete-case and model-specific exclusion approaches for handling missing data, both of which yielded consistent findings. Therefore, the complete-case approach was adopted for the main analyses. Collinearity among variables was assessed using variance inflation factors (VIF), which ranged from 1.74 to 4.64, indicating low to moderate multicollinearity. All statistical analysis was conducted in Stata 15.0.

## 3. Results

Table 1 summarizes the demographic, clinical characteristics, and mortality of patients stratified by malnutrition status and age groups (young-old: 65–84 years and old-old: ≥85 years). Among the 2677 participants, 59.3% (1587) were young-old, and 40.7% (1090) were old-old. The overall prevalence of patients at risk of malnutrition was 29.2%, significantly higher in the old-old (37.1%) compared to the young-old (23.8%). In both groups at risk of malnutrition, there was a significantly higher prevalence of pneumonia (young-old: 34.1% vs. 23.6%, old-old: 37.1% vs. 29.2%), and hyponatremia (young-old: 20.1% vs. 13.0%, old-old: 27.5% vs. 16.2%).

Regardless of age, there was a significantly higher prevalence of severe and terminal frailty (CFS 7–9) in the malnutrition group (young-old: 36.8% vs. 19.8%, old-old: 51.5% vs. 29.0%). In the malnutrition group, there was a significantly higher proportion of patients who were dependent or required assistance in all the ADL items (young-old: 47.1% vs. 21.8%, old-old: 59.7% vs. 35.6%). In the old-old group, at the risk of malnutrition, there was a higher prevalence of frailty based on HFRS scores (12.6% vs. 8.0%). The 30-day and 90-day mortality rates were significantly higher in the groups at risk of malnutrition (young-old group: 30-day—11.4% vs. 3.8% and 90-day—19.6% vs. 7.5%; old-old group: 30-day 21.5% vs. 8.0% and 90-day—29.2% vs. 13.0%).

**Figure 1:**
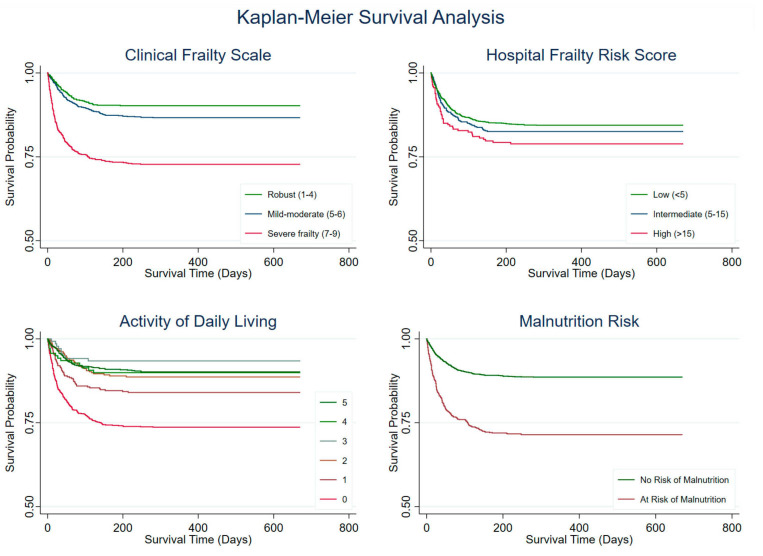
Kaplan–Meier curves based on Clinical Frailty Scale, Hospital Frailty Risk Score, Activity of Daily Living, and Malnutrition Risk.

The probability of survival over time stratified by groups for incremental CFS, HFRS, and ADL scores is shown using the Kaplan–Meier survival curves (Figure 1). Significant differences in survival distributions were observed for CFS ($\chi^{2}$ = 109.76, *p* < 0.001), ADL scores ($\chi^{2}$ = 116.75, *p* < 0.001), and risk of malnutrition ($\chi^{2}$ = 127.00, *p* < 0.001). Patients with higher CFS scores had notably lower survival probabilities, particularly for those classified as severe/terminal frailty (CFS ≥ 7). The ADL score = 0, indicating fully functional dependence/assistance needed, and was associated with decreased survival probabilities.

There was a significant association of 30-day mortality with higher CFS scores (aOR = 1.498, 95% CI: 1.349–1.664, *p* < 0.001) (Table 2), higher HFRS scores (aOR = 1.020, 95% CI: 1.001–1.040, *p* = 0.038), and lower ADL scores (or better functional status) (aOR = 0.819, 95% CI: 0.753–0.890, *p* < 0.001). Risk of malnutrition demonstrated the strongest association across all models (ADL model: aOR = 2.573, 95% CI: 1.922–3.443, *p* < 0.001; CFS model: aOR = 2.348, 95% CI: 1.738–3.156, *p* < 0.001; HFRS model: aOR = 2.944, 95% CI: 2.210–3.922, *p* < 0.001).

Unlike HFRS, both CFS (aOR = 1.361, 95% CI: 1.255–1.475, *p* < 0.001) and ADL (aOR = 0.837, 95% CI: 0.785–0.892, *p* < 0.001) were significantly associated with 90-day mortality. Risk of malnutrition remained significantly associated with mortality risk (ADL: aOR = 2.422, 95% CI: 1.908–3.075, *p* < 0.001; CFS: aOR = 2.320, 95% CI: 1.824–2.951, *p* < 0.001; HFRS: aOR = 2.770, 95% CI: 2.192–3.500, *p* < 0.001).

**Figure 2:**
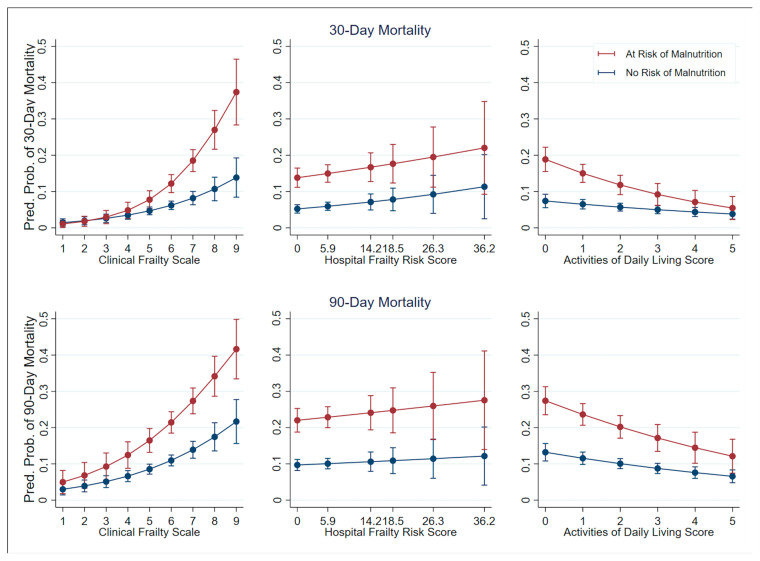
Margins Plot on Predicted Mortality Probability by Frailty, Activity of Daily Living, and Malnutrition Risk. The figure corresponds to the results in Table 3, which are adjusted by demographics and Charlson Comorbidity Index.

Table 3 and Figure 2 summarize the multivariate logistic regression results incorporating interaction terms of malnutrition risk with either CFS, HFRS, or ADL. The CFS score remained a significant predictor for mortality (30-day mortality: aOR = 1.359, 95% CI: 1.181–1.564, *p* < 0.001; 90-day: aOR = 1.326, 95% CI: 1.064–1.440, *p* < 0.001), with the interaction term between CFS and malnutrition approaching significance for 30-day mortality (aOR = 1.236, 95% CI: 0.999–1.530, *p* = 0.051). For the HFRS model, the interaction term was not significant, though malnutrition remained an independent predictor of mortality (30-day: aOR = 3.048, 95% CI: 2.177–4.267, *p* < 0.001; 90-day: aOR = 2.752, 95% CI: 2.099–3.609, *p* < 0.001). In the ADL model, higher ADL scores were associated with lower mortality (30-day: aOR = 0.866, 95% CI: 0.780–0.962, *p* = 0.007; 90-day: aOR = 0.853, 95% CI: 0.787–0.924, *p* < 0.001), with no significant interaction with malnutrition observed. Figure 2 illustrates that mortality probabilities changed sharply with higher CFS/ADL scores and malnutrition.

Table 4 presents the subgroup analysis for the old-old patient subgroup with cognitive impairment and/or dementia. For 30-day mortality, the CFS score (aOR = 1.239, 95% CI: 1.027–1.493, *p* = 0.025) and its interaction with malnutrition risk (aOR = 1.435, 95% CI: 1.082–1.902, *p* = 0.012) were significant. For 90-day mortality, similar patterns were observed, with the CFS score (aOR = 1.238, 95% CI: 1.064–1.440, *p* = 0.006) and its interaction with malnutrition risk (aOR = 1.263, 95% CI: 1.005–1.588, *p* = 0.045) remaining significant. Malnutrition risk was significantly associated with higher mortality, whereas higher ADL scores (aOR = 0.775, 95% CI: 0.678–0.887, *p* < 0.001) were protective.

## 4. Discussion

Singapore has one of the fastest-growing populations in the world, where one in four of the population will be ≥65 years old in 2030, and almost half are projected to be either pre-frail or frail [7,40]. In our study population, three-quarters had at least mild frailty, but the prevalence increased significantly with age, especially in those who were at risk of malnutrition, to almost nine in ten in the old-old. A similar number of the old-old, within the group at risk of malnutrition, had at least one ADL decline. More than half of the young-old and three-quarters of the old-old non-malnourished had at least one ADL decline. Frailty remission has been shown to be associated with reduced risk of developing future dementia [41].

Malnutrition risk was independently associated with both 30- and 90-day mortality in multivariable analysis, and this association was more pronounced among individuals with higher frailty severity or ADL dependency. Malnutrition is widely recognized as a modifiable risk factor for cognitive impairment, frailty, mortality, and other adverse outcomes [42,43]. In this study, the prevalence of malnutrition risk in older adults with dementia or cognitive impairment was almost one third, which is slightly lower than reported in a recent meta-analysis by Arifin et al., which reported a prevalence of 32.5% and a further 46.8% at risk of malnutrition [44]. The pooled prevalence in those living in long-term care is reported to be 57.4% [45]. Amongst the old-old with malnutrition risk in our study population, slightly more than half had severe frailty, and almost two-thirds were dependent in all ADLs. Regardless of dementia, those with ADL dependency and malnutrition have shown to have an increased mortality rate [21]. Older adults with declining cognition and functional status need to be screened early for malnutrition risk, as studies have shown that optimizing nutritional status will optimize functional status, and in turn may reduce dementia risk [41,46]. However, the effectiveness of intervention is closely linked to timing, with earlier nutritional support possibly offering greater potential to mitigate adverse outcomes [19].

While CFS, HFRS, and ADL were significantly associated with 30-day mortality, HFRS showed no significant association with 90-day mortality in multivariable analysis. The relationship between CFS and all-cause mortality has been extensively examined, with prior studies demonstrating that severe frailty and cognitive impairment were associated with significantly reduced survival rates [47]. Research focusing on hospitalized patients has shown that higher CFS scores, particularly those with CFS 7–8, were strong independent predictors of survival time regardless of their acute illness [48]. There are also some studies demonstrating that both ADL and instrumental ADL significantly influenced all-cause mortality [15,21,49,50]. However, research specifically focusing on the relationship between ADL and mortality in patients with dementia and cognitive impairment remains limited.

Studies investigating the association between HFRS and mortality yielded mixed results in our study population. One possible reason for this is that HFRS relies on ICD-10 diagnostic codes from the current hospitalization; thus, only clearly documented diagnoses are captured, overlooking mild or moderate malnutrition. As an indicator reflecting comorbidity-driven frailty, HFRS does not adequately capture functional status. Furthermore, information on comorbidities may not be available on admission, and HFRS may underestimate frailty status if the patient is not known to the healthcare system.

In models, CFS showed the strongest association, especially in the old-old group with malnutrition risk, whereas HFRS was the weakest. These findings are similar to those in previous research [51]. Incorporating malnutrition risk into each model improved their performance, indicating its unique contribution to building associations with mortality. In the old-old patient group, malnutrition risk significantly amplified the mortality risk in those with severe frailty (high CFS scores). Both cognitive frailty and physical frailty were associated with malnutrition among Turkish older adults, but cognitive frailty showed a stronger link [52]. Malnutrition accelerates cognitive decline through multiple biological mechanisms. Firstly, malnutrition can impair neurotransmitter synthesis, reduce brain energy supply, and enhance chronic inflammation and oxidative stress; these factors collectively damage neurons, decrease brain plasticity, and hasten cognitive deterioration [25,53,54]. As cognitive decline progresses, individuals often experience worsening self-care abilities, increased feeding difficulties, and reduced physical activity, all of which exacerbate malnutrition and contribute to a vicious cycle of further functional and cognitive deterioration [25,55,56]. Low levels of vitamin D, micronutrients, decreased albumin, and deficiency of antioxidants are among the key mechanisms through which malnutrition may contribute to the progression of frailty and cognitive impairment [57]. Additionally, the prevalence of delirium was highest among old-old individuals at risk of malnutrition (35.2%), compared to non-malnourished old-old (26.7%) and young-old individuals at risk of malnutrition (28.0%), suggesting that factors beyond age, such as malnutrition risk, may further predispose this group to delirium.

The strength of our study includes a robust database, with comprehensive data on ADL, HFRS, nutrition, and CFS obtained from electronic medical records. However, several limitations warrant mention. First, the accuracy of data obtained from the database is dependent on the accuracy of data entry and data coding. Second, as the inclusion criteria were limited to those with either primary or secondary diagnoses of dementia or cognitive impairment, the study findings cannot be generalized to other older patients. Third, this study was based on cross-sectional data collected during admission. There may be recall bias. Fourth, we had no information on factors which may impact mortality, such as proper care transition, food insecurity, compliance with nutritional supplements post-discharge, and advance care planning. Finally, we could only determine association with risk of malnutrition using the 3-MinNS. Determining malnutrition using the GLIM criteria could have provided better diagnostic accuracy. The 3-MinNS has been validated in hospitalized older adults locally, and its components such as unintentional weight loss, reduced intake, and muscle wasting align with key phenotypic and etiologic criteria of the GLIM framework [58]. It has demonstrated good diagnostic performance, with a reported sensitivity of 86% and specificity of 83% for identifying malnutrition, and a sensitivity of 93% and specificity of 86% for detecting severe malnutrition [39]. Additionally, our dataset did not include the cause of death, which limits our ability to draw causal inferences about the pathways linking nutritional risk to mortality outcomes.

## 5. Conclusions

For older patients with dementia or cognitive impairment, CFS and ADL demonstrated superior association with 30- and 90-day mortality compared to HFRS. Risk of malnutrition was independently associated with mortality and modified the association between frailty and mortality, particularly among adults aged ≥85 years.

## Figures and Tables

**Table 1:** Demographic and Clinical Characteristics of.

		65–84 Years Old 1587 (59.3)			≥85 Years Old 1090 (40.7)		
Malnutrition Risk							
		No	Yes		No	Yes	
	N = 2677	1209 (76.2)	378 (23.8)	*p* Value	686 (62.9)	404 (37.1)	*p* Value
Sex				0.410			0.710
Male	1253 (46.8)	637 (52.7)	190 (50.3)		271 (39.5)	155 (38.4)	
Female	1424 (53.2)	572 (47.3)	188 (49.7)		415 (60.5)	249 (61.6)	
Race				0.282			0.424
Chinese	2180 (81.4)	945 (78.2)	304 (80.4)		591 (86.2)	340 (84.2)	
Indian	159 (5.9)	81 (6.7)	21 (5.6)		35 (5.1)	22 (5.5)	
Malay	204 (7.6)	117 (9.9)	27 (7.1)		32 (4.7)	28 (6.9)	
Others	134 (5.0)	66 (5.5)	26 (6.9)		28 (4.1)	14 (3.5)	
Comorbidities							
Diabetes Mellitus	1129 (42.2)	605 (50.0)	150 (39.7)	<0.001	243 (35.4)	131 (32.4)	0.314
Hypertension	1479 (55.3)	750 (62.0)	181 (47.9)	<0.001	356 (51.9)	192 (47.5)	0.163
Hyperlipidemia	1108 (41.4)	600 (49.6)	138 (36.5)	<0.001	257 (37.5)	113 (28.0)	0.001
Discharge Diagnosis							
Delirium	723 (27.0)	292 (24.2)	106 (28.0)	0.128	183 (26.7)	142 (35.2)	0.003
Pneumonia	764 (28.5)	285 (23.6)	129 (34.1)	<0.001	200 (29.2)	150 (37.1)	0.006
UTI	771 (28.8)	321 (26.6)	117 (31.0)	0.095	207 (30.2)	126 (31.2)	0.726
Constipation	151 (5.6)	72 (6.0)	19 (5.0)	0.498	42 (6.1)	18 (4.5)	0.244
Hyponatremia	455 (17.0)	157 (13.0)	76 (20.1)	0.001	111 (16.2)	111 (27.5)	<0.001
Ischemic Stroke	220 (8.2)	119 (9.8)	23 (6.1)	0.025	52 (7.6)	26 (6.4)	0.479
Intracranial Bleed	115 (4.3)	66 (5.5)	10 (2.7)	0.025	20 (2.9)	19 (4.7)	0.125
Acute Myocardial Infarction	257 (9.6)	114 (9.4)	27 (7.1)	0.173	56 (8.2)	60 (14.9)	0.001
Heart failure	186 (7.0)	88 (7.3)	15 (4.0)	0.023	63 (9.2)	20 (5.0)	0.011
Orthostatic Hypotension	174 (6.5)	86 (7.1)	32 (8.5)	0.382	37 (5.4)	19 (4.7)	0.618
Osteoporosis Fractures	140 (5.2)	53 (4.4)	15 (4.0)	0.728	56 (8.2)	16 (4.0)	0.007
Parkinson’s Disease	120 (4.5)	60 (5.0)	24 (6.4)	0.293	23 (3.4)	13 (3.2)	0.904
Sepsis	327 (12.2)	139 (11.5)	57 (15.1)	0.065	72 (10.5)	59 (14.6)	0.044
CCI, median (IQR)	6 (4–7)	6 (4–8)	5.5 (4–7)	0.442	5 (4–7)	5 (4–7)	0.216
CFS				<0.001			<0.001
1–4	758 (28.3)	434 (35.9)	83 (22.0)		183 (26.7)	58 (14.4)	
5–6	1134 (42.4)	536 (44.3)	156 (41.3)		304 (44.3)	138 (34.2)	
7–9	785 (29.3)	239 (19.8)	139 (36.8)		199 (29.0)	208 (51.5)	
HFRS				0.609			0.024
0–4	1963 (73.3)	898 (74.3)	271 (71.7)		516 (75.2)	278 (68.8)	
5–15	487 (18.2)	221 (18.3)	76 (20.1)		115 (16.8)	75 (18.6)	
16-	227 (8.5)	90 (7.4)	31 (8.2)		55 (8.0)	51 (12.6)	
ADL				<0.001			<0.001
0	927 (34.6)	264 (21.8)	178 (47.1)		244 (35.6)	241 (59.7)	
1	364 (13.6)	144 (11.9)	51 (13.5)		118 (17.2)	51 (12.)	
2	317 (11.8)	151 (12.5)	37 (9.8)		88 (12.8)	41 (10.2)	
3	137 (5.1)	70 (5.8)	20 (5.3)		36 (5.3)	11 (2.7)	
4	139 (5.2)	84 (7.0)	11 (2.9)		34 (5.0)	10 (2.5)	
5	793 (29.6)	496 (41.0)	81 (21.4)		166 (24.2)	50 (12.4)	
Mortality (30 day and 90 day)							
30-day mortality	231 (8.6)	46 (3.8)	43 (11.4)	<0.001	55 (8.0)	87 (21.5)	<0.001
90-day mortality	372 (13.9)	91 (7.5)	74 (19.6)	<0.001	89 (13.0)	118 (29.2)	<0.001

CFS: Clinical Frailty Score; ADL: Activity of Daily Living; HFRS: Hospital Frailty Risk Score; CCI: Charlson Comorbidity Index.

**Table 2:** Multivariate Logistic Regression Results for 30-Day and 90-Day Mortality: (**A**) Clinical Frailty Score; (**B**) Hospital Frailty Risk Score; (**C**) Activity of Daily Living.

(A) Clinical Frailty Score (CFS)						
	30-Day Mortality			90-Day Mortality		
Variable	aOR (95% CI)	SE	*p*-Value	OR (95% CI)	SE	*p*-Value
CFS score	1.498 (1.349, 1.664)	0.080	<0.001	1.361 (1.255, 1.475)	0.056	<0.001
Malnutrition	2.348 (1.748, 3.156)	0.034	<0.001	2.320 (1.824, 2.951)	0.029	<0.001
**(B) Hospital Frailty Risk Score (HFRS)**						
HFRS score	1.020 (1.001, 1.040)	0.010	0.038	1.008 (0.992, 1.025)	0.008	0.333
Malnutrition	2.944 (2.210, 3.922)	0.431	<0.001	2.770 (2.192, 3.500)	0.331	<0.001
**(C) Activity of Daily Living (ADL)**						
ADL (number)	0.819 (0.753, 0.890)	0.035	<0.001	0.837 (0.785, 0.892)	0.027	<0.001
Malnutrition	2.573 (1.922, 3.443)	0.383	<0.001	2.422 (1.908, 3.075)	0.295	<0.001

aOR: adjusted Odd Ratio; CI: Confidence Interval; SE: Standard Error; Models were adjusted by demographics and Charlson Comorbidity Index. Malnutrition means at risk of malnutrition.

**Table 3:** Multivariate Logistic Regression Results with interaction. (**A**) Clinical Frailty Score; (**B**) Hospital Frailty Risk Score; (**C**) Activity of Daily Living.

(A) Clinical Frailty Score (CFS)						
	30-Day Mortality			90-Day Mortality		
Variable	aOR (95% CI)	SE	*p*-Value	OR (95% CI)	SE	*p*-Value
CFS score	1.359 (1.181, 1.564)	0.097	<0.001	1.326 (1.191, 1.476)	0.072	<0.001
Malnutrition risk	0.603 (0.148, 2.451)	0.431	0.479	1.613 (0.576, 4.519)	0.848	0.363
CFS × Malnutrition	1.236 (0.999, 1.530)	0.134	0.051	1.061 (0.902, 1.247)	0.088	0.476
**(B) Hospital Frailty Risk Score (HFRS)**						
HFRS score	1.024 (0.996, 1.054)	0.015	0.095	1.007 (0.984, 1.031)	0.012	0.548
Malnutrition risk	3.048 (2.177, 4.267)	0.523	<0.001	2.752 (2.099, 3.609)	0.381	<0.001
HFRS × Malnutrition	0.993 (0.956, 1.031)	0.019	0.699	1.002 (0.969, 1.035)	0.017	0.927
**(C) Activity of Daily Living (ADL)**						
ADL score	0.866 (0.780, 0.962)	0.046	0.007	0.853 (0.787, 0.924)	0.035	<0.001
Malnutrition risk	3.053 (2.134, 4.369)	0.558	<0.001	2.597 (1.930, 3.493)	0.393	<0.001
ADL × Malnutrition	0.864 (0.725, 1.029)	0.077	0.101	0.949 (0.832, 1.083)	0.064	0.437

aOR: adjusted Odd Ratio; CI: Confidence Interval; SE: Standard Error. Models were adjusted by demographics and Charlson Comorbidity Index. Malnutrition means risk of malnutrition.

**Table 4:** Multivariate Logistic Regression Results with interaction (≥85 Years). (**A**) Clinical Frailty Score; (**B**) Hospital Frailty Risk Score; (**C**) Activity of Daily Living.

(A) Clinical Frailty Score (CFS)						
	30-Day Mortality			90-Day Mortality		
Variable	aOR (95% CI)	SE	*p* Value	OR (95% CI)	SE	*p* Value
CFS score	1.239 (1.027, 1.493)	0.118	0.025	1.238 (1.064, 1.440)	0.096	0.006
Malnutrition risk	0.217 (0.033, 1.416)	0.208	0.110	0.483 (0.109, 2.145)	0.367	0.339
CFS × Malnutrition *	1.435 (1.082, 1.902)	0.206	0.012	1.263 (1.005, 1.588)	0.148	0.045
**(B) Hospital Frailty Risk Score (HFRS)**						
HFRS score	1.019 (0.979, 1.061)	0.021	0.362	1.007 (0.972, 1.042)	0.018	0.702
Malnutrition risk	2.965 (1.915, 4.589)	0.661	<0.001	2.564 (1.772, 3.712)	0.484	<0.001
HFRS × Malnutrition *	0.992 (0.943, 1.044)	0.026	0.753	1.003 (0.959, 1.049)	0.023	0.904
**(C) Activity of Daily Living (ADL)**						
ADL score	0.791 (0.670, 0.935)	0.067	0.006	0.775 (0.678, 0.887)	0.053	<0.001
Malnutrition risk	2.633 (1.686, 4.110)	0.598	<0.001	2.330 (1.588, 3.419)	0.456	<0.001
ADL × Malnutrition *	0.946 (0.736, 1.217)	0.122	0.669	0.961 (0.779, 1.185)	0.103	0.708

aOR: adjusted Odd Ratio; CI: Confidence Interval; SE: Standard Error. Models were adjusted by demographics and Charlson Comorbidity Index. * risk of malnutrition.

## Data Availability

The patient-level datasets generated and/or analyzed during the current study are not publicly available due to privacy considerations but can be obtained from the corresponding author upon reasonable request.

## Supplementary Materials

The following supporting information can be downloaded at: https://www.mdpi.com/article/10.3390/nu17162612/s1, Table S1: Multivariate Logistic Regression Results for 30-Day and 90-Day Mortality (Patient Level). Table S2: Multivariate Logistic Regression Results with interaction (Patient Level). Table S3: Subgroup Analysis Based on Age (≥85 Years) (Patient Level).
