# Supplementary material for: The Impact of Frailty, Activity of Daily Living, and Malnutrition on Mortality in Older Adults with Cognitive Impairment and Dementia
Source: Nutrients. 2025 Aug 12;17(16):2612. doi: 10.3390/nu17162612 (PMC12389014; doi:10.3390/nu17162612)
Supplement: Supplementary file 1 [file nutrients-17-02612-s001.zip › nutrients-3776684-supplementary.pdf]

Additional file

Sensitivity analysis

TableS1 Multivariate Logistic Regression Results for 30-Day and 90-Day Mortality (Patient Level)

Variable	*aOR (95%CI)*	*SE*	*p*-value	Variable	*aOR (95%CI)*	*SE*	*p*-value	Variable	*aOR (95%CI)*	*SE*	*p*-value
**Outcome 1: 30-day mortality**											
**CFS**				**HFRS**				**ADL**			
**CFS score**	**1.475(1.309,1.661)**	**0.089**	**<0.001**	HFRS score	1.012(0.990,1.034)	0.011	0.294	**ADL score**	**0.829(0.757,0.908)**	**0.038**	**<0.001**
**Malnutrition**	**2.113(1.509,2.959)**	**0.363**	**<0.001**	**Malnutrition**	**2.677(1.931,3.711)**	**0.446**	**<0.001**	**Malnutrition**	**2.344(1.682,3.267)**	**0.397**	**<0.001**
**Outcome 2: 90-day mortality**											
**CFS**				**HFRS**				**ADL**			
**CFS score**	**1.293(1.181,1.415)**	**0.060**	**<0.001**	HFRS score	1.001(0.982,1.020)	0.010	0.897	**ADL score**	**0.835(0.778,0.897)**	**0.030**	**<0.001**
**Malnutrition**	**2.257(1.716,2.968)**	**0.315**	**<0.001**	**Malnutrition**	**2.660(2.036,3.473)**	**0.362**	**<0.001**	**Malnutrition**	**2.317(1.765,3.040)**	**0.321**	**<0.001**

aOR: adjusted Odd Ratio; CI: Confidential Interval; SE: Standard Error; CFS: Clinical Frailty Scale; HFRS: Hospital Frailty Risk Score; ADL: Activity of Daily Living

Models were adjusted by demographics and Charlson Comorbidity Index (CCI)

TableS2 Multivariate Logistic Regression Results with interaction (Patient Level)

**Outcome 1: 30-day mortality**											
**CFS**				**HFRS**				**ADL**			
**Variable**	***OR (95%CI)***	***SE***	***P***	**Variable**	***OR (95%CI)***	***SE***	***P***	**Variable**	***OR (95%CI)***	***SE***	***P***
**CFS score**	**1.357(1.162,1.584)**	**0.107**	**<0.001**	HFRS score	1.018(0.985,1.052)	0.015	0.290	**ADL score**	**0.854(0.760,0.958)**	**0.050**	**0.007**
Malnutrition	0.614(0.125,3.012)	0.498	0.479	**Malnutrition**	**2.802(1.919,4.090)**	**0.541**	**<0.001**	**Malnutrition**	**2.580(1.709,3.897)**	**0.543**	**<0.001**
Interaction	1.213(0.952,1.545)	0.150	0.117	Interaction	0.990(0.947,1.034)	0.022	0.640	Interaction	0.929(0.771,1.119)	0.088	0.438
**Outcome 2: 90-day mortality**											
**CFS**				**HFRS**				**ADL**			
**Variable**	***OR (95%CI)***	***SE***	***P***	**Variable**	***OR (95%CI)***	***SE***	***P***	**Variable**	***OR (95%CI)***	***SE***	***P***
**CFS score**	**1.274(1.132,1.434)**	**0.077**	**<0.001**	HFRS score	1.006(0.979,1.034)	0.014	0.667	**ADL score**	**0.829(0.757,0.907)**	**0.038**	**<0.001**
**Malnutrition**	**1.832(0.586,5.725)**	**1.065**	**0.298**	**Malnutrition**	**2.756(2.029,3.743)**	**0.430**	**<0.001**	**Malnutrition**	**2.250(1.599,3.168)**	**0.393**	**<0.001**
Interaction	1.035(0.863,1.241)	0.096	0.712	Interaction	0.991(0.954,1.029)	0.019	0.642	Interaction	1.020(0.884,1.177)	0.074	0.784

OR: Odd Ratio; CI: Confidential Interval; SE: Standard Error; CFS: Clinical Frailty Scale; HFRS: Hospital Frailty Risk Score; ADL: Activity of Daily Living Models were adjusted by demographics and Charlson Comorbidity Index (CCI). Interaction is the interaction term between CFS/HFRS/ADL score and Malnutrition.

TableS3 Subgroup Analysis Based on Age (≥85 Years) (Patient Level)

**Outcome1: 30days mortality**											
**CFS**				**HFRS**				**ADL**			
**Variable**	***OR (95%CI)***	***SE***	***P***	**Variable**	***OR (95%CI)***	***SE***	***P***	**Variable**	***OR (95%CI)***	***SE***	***P***
**CFS score**	**1.275(1.036,1.569)**	**0.135**	**0.022**	**HFRS score**	**1.000(0.952,1.050)**	**0.025**	**1.000**	**ADL score**	**0.836(0.702,0.995)**	**0.074**	**0.044**
**Malnutrition**	**0.223(0.025,1.990)**	**0.249**	**0.179**	**Malnutrition**	**2.669(1.632,4.362)**	**0.669**	**<0.001**	**Malnutrition**	**2.421(1.438,4.078)**	**0.644**	**<0.001**
**Interaction**	**1.401(1.011,1.942)**	**0.233**	**0.043**	**Interaction**	**0.998(0.939,1.060)**	**0.031**	**0.940**	**Interaction**	**0.968(0.746,1.255)**	**0.128**	**0.804**
**Outcome2: 90days mortality**											
**CFS**				**HFRS**				**ADL**			
**Variable**	***OR (95%CI)***	***SE***	***P***	**Variable**	***OR (95%CI)***	***SE***	***P***	**Variable**	***OR (95%CI)***	***SE***	***P***
**CFS score**	**1.207(1.019,1.429)**	**0.104**	**0.029**	**HFRS score**	**0.999(0.958,1.041)**	**0.021**	**0.946**	**ADL score**	**0.802(0.692,0.929)**	**0.060**	**<0.003**
**Malnutrition**	**0.573(0.107,3.074)**	**0.491**	**0.516**	**Malnutrition**	**2.637(1.729,4.022)**	**0.568**	**<0.001**	**Malnutrition**	**2.322(1.483,3.636)**	**0.531**	**<0.001**
**Interaction**	**1.230(0.949,1.594)**	**0.163**	**0.117**	**Interaction**	**0.996(0.945,1.049)**	**0.027**	**0.877**	**Interaction**	**0.969(0.774,1.214)**	**0.111**	**0.787**

OR: Odd Ratio; CI: Confidential Interval; SE: Standard Error; CFS: Clinical Frailty Scale; HFRS: Hospital Frailty Risk Score; ADL: Activity of Daily Living Models were adjusted by demographics and Charlson Comorbidity Index (CCI).. Interaction is the interaction term between CFS/HFRS/ADL score and Malnutrition.
